# Supplementary material for: Single-molecule analysis of steroid receptor and cofactor action in living cells
Source: Nat Commun. 2017 Jun 21;8:15896. doi: 10.1038/ncomms15896 (PMC5482060; doi:10.1038/ncomms15896)
Supplement: Supplementary Information [file ncomms15896-s1.pdf]

Type of file: PDF
Size of file: 0 KB
Title of file for HTML: Supplementary Information
Description: Supplementary Figures and Supplementary Table

Type of file: XLSX
Size of file: 0 KB
Title of file for HTML: Supplementary Data 1
Description: Number of cells and tracks analyzed and residence times and bound fractions of tested factors. The data shows several parameters used during single-molecule tracking for all the tested proteins. For more details, see Methods.

Type of file: MP4
Size of file: 0 KB
Title of file for HTML: Supplementary Movie 1
Description: Single-Molecule Tracking Data of activated HaloTag-GR in 3617 Cells. A time-lapse sequence of single GR molecules captured at different interval acquisition times: 15ms (left), 200ms (middle), or 2000ms (right). Movie plays in real time. Nuclear boundaries are shown as red, non-continuous lines. The movie illustrates how different interval times sample different population of molecules. During fast acquisition rates (left), diffusing molecules are easily identified while long-lived bound molecules are rapidly lost due to photobleaching. At the lowest acquisition rate (right), only bound molecules can be discriminated.

Type of file: PDF
Size of file: 0 KB
Title of file for HTML: Peer Review File
Description:

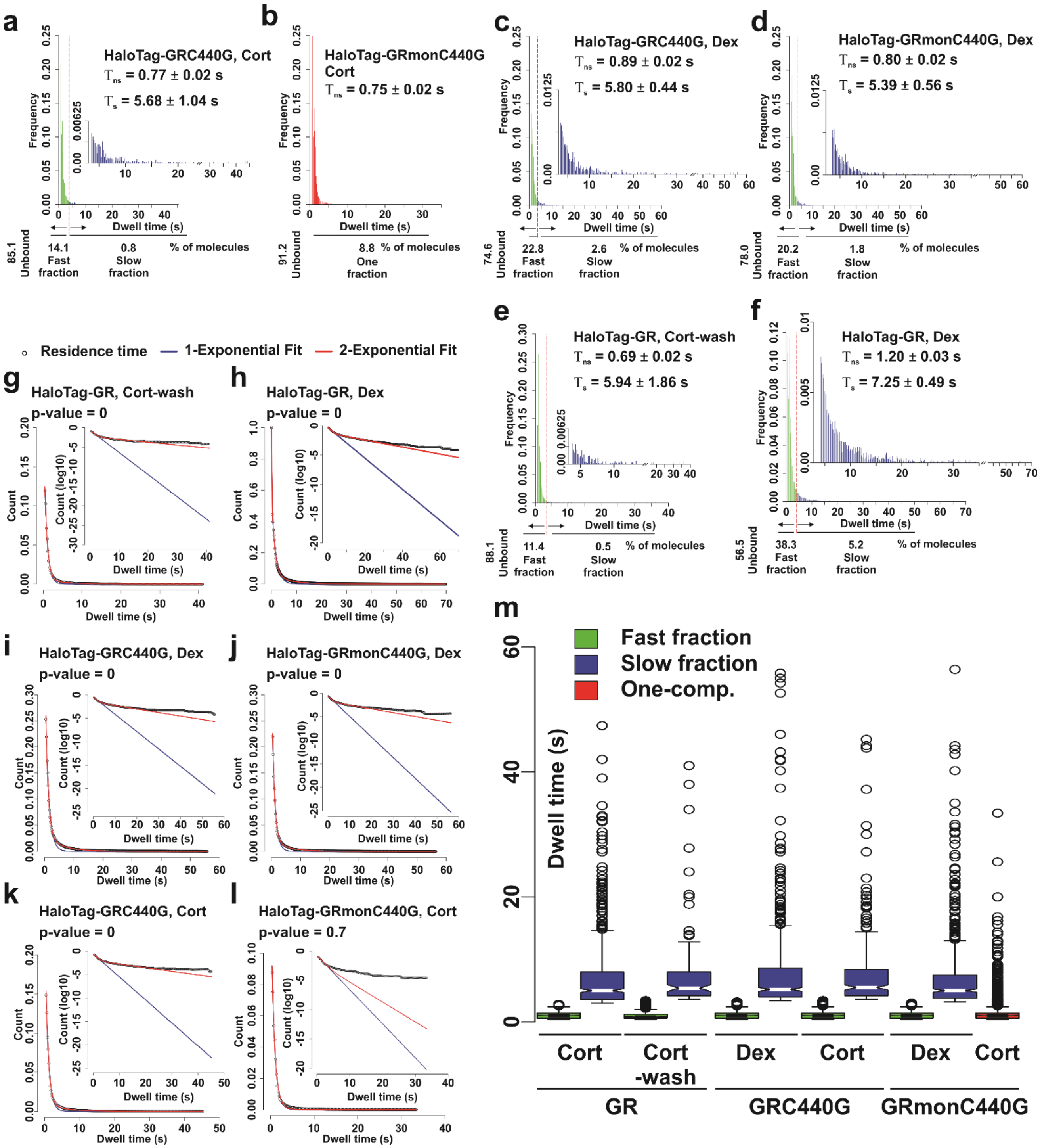

Supplementary Figure 1. **HaloTag-GR Single-molecule tracking data with different treatments and DNA binding mutants.** (a-f) Histograms represent the distribution of residence times. Red dashed line separates the fast short-lived fraction (green bars) from the slow long-lived fraction (blue bars). Expanded view represents slow long-lived fraction of the residence time distribution. The average residence time of fast short-lived (T_{ns}) and slow long-lived (T_s) fraction are presented in the histogram. The percentage of molecules unbound, bound at the fast short-lived fraction, and bound at the slow long-lived fraction is presented below the histogram. Cort, corticosterone. Wash indicates withdraw of the ligand by multiple washouts. (b) GRmonC440G histogram with Cort treatment indicates only one-component (red bars). (g-l) Data represents collected tracks (black circles) in a survival distribution plot, showing the single-molecule residence times fitted to a single- (blue line) or double-exponential (red line) decay model. Expanded view represents the single-molecule residence time fitted to a single- (blue line) or double-exponential (red line) decay model with y-axis plotted as a log10. F-test determines the statistical significance of the fit for each treatment condition. (m) Box-plot represents the distribution of dwell times for all molecules in the fast short-lived (green boxes) and slow long-lived (blue boxes) fraction for GR. GRmonC440G in Cort treated cells is presented as one-component fraction (red boxes). Box-plot for GR in Dex treated cells is represented in Supplementary Fig. 3i. Statistical outliers are shown as black circles. Exposure time 10 ms; interval time 200 ms.

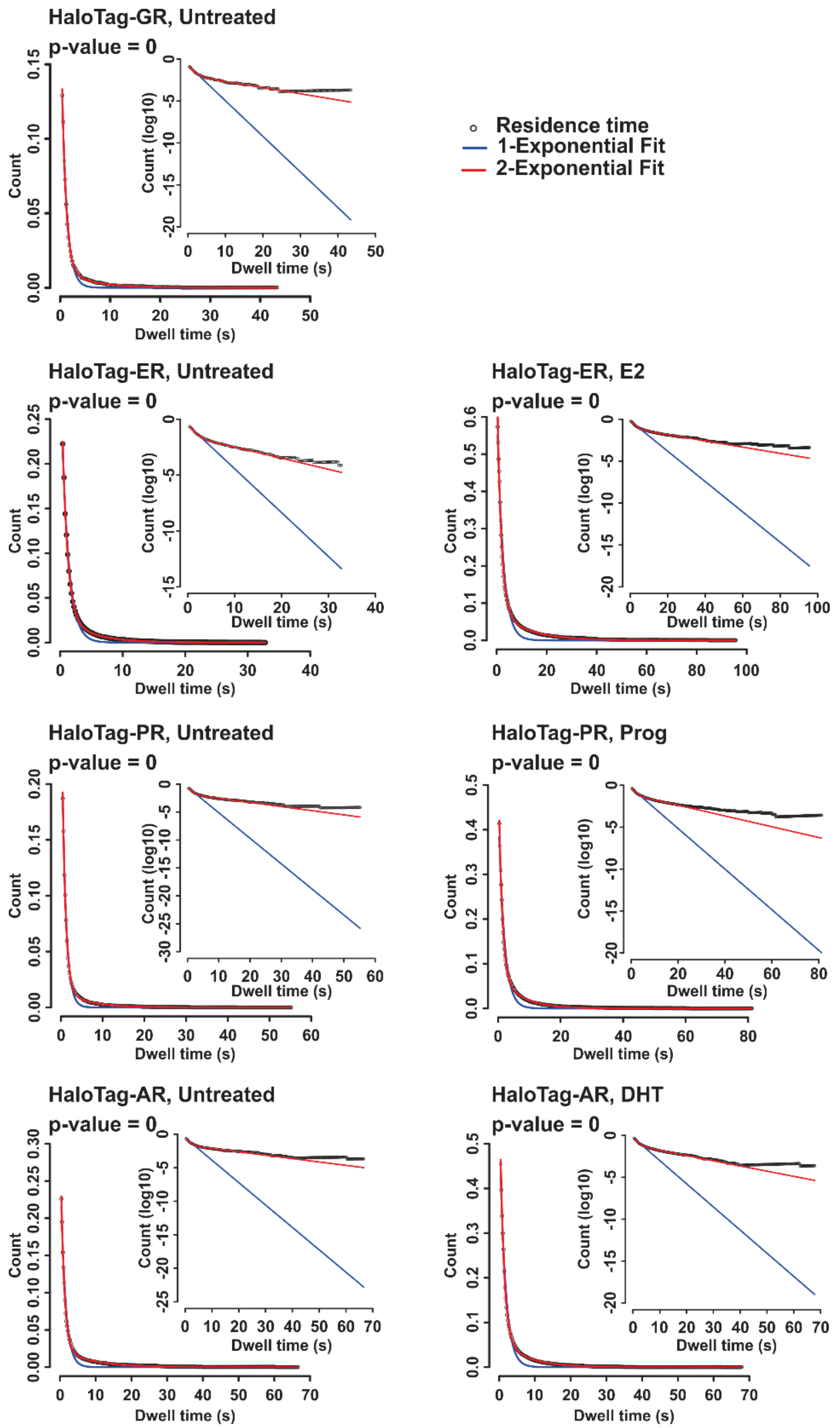

Supplementary Figure 2. **Exponential fitting of HaloTag-ER, HaloTag-GR, HaloTag-PR, and HaloTag-AR Single-molecule tracking data.** Indicated data represents collected tracks (black circles) showing the single-molecule residence times fitted to a single- (blue line) or double-exponential (red line) decay model. Expanded view represents the single-molecule residence time fitted to a single- (blue line) or double-exponential (red line) decay model with y-axis plotted as a log10. F-test determines the statistical significance of the fit for each treatment condition. Exponential fitting of GR in Dex treated cells is represented in Supplementary Fig. 1h. Exposure time 10 ms; interval time 200 ms.

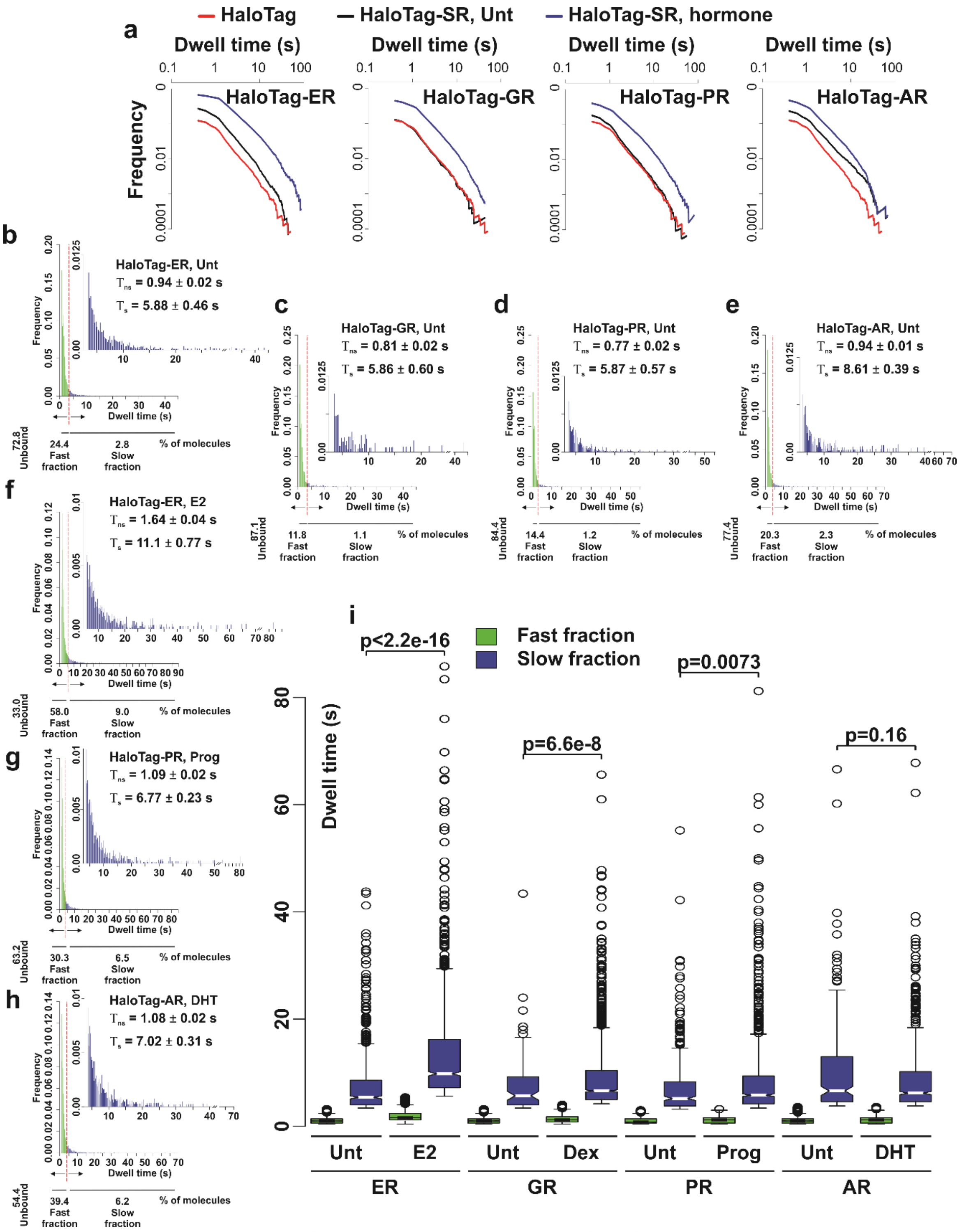

Supplementary Figure 3. HaloTag-ER, HaloTag-GR, HaloTag-PR, and HaloTag-AR Single-molecule tracking data. (a) Comparison of dwell time distribution between HaloTag alone (red line), steroid receptor (SR) in untreated (black line), and SR in hormone treated (blue line) cells. Both axes are plotted as a log10. (b-h) Histograms represent the distribution of residence times. Red dashed line separates the fast short-lived fraction (green bars) from the slow long-lived fraction (blue bars). Expanded view represents slow long-lived fraction of the residence time distribution. The average residence time of fast short-lived (T_{ns}) and slow long-lived (T_s) fraction are presented in the histogram. The percentage of molecules unbound, bound at the fast short-lived fraction, and bound at the slow long-lived fraction is presented below the histogram for ER (b), GR (c), PR (d), and AR (e) untreated, and ER E2 treated (f), PR Prog treated (g), and AR DHT treated (h). GR Dex treated histogram is represented in Supplementary Fig. 1f. (i) Box-plot represents the distribution of dwell times for all molecules in the fast short-lived (green boxes) and slow long-lived (blue boxes) fraction for ER untreated and E2 treated, GR untreated and Dex treated, PR untreated and Prog, AR untreated and DHT treated cells. Statistical outliers are shown as black circles. p-values represent a Two-sample Kolmogorov Smirnov test defined by the brackets. Exposure time 10 ms; interval time 200 ms.

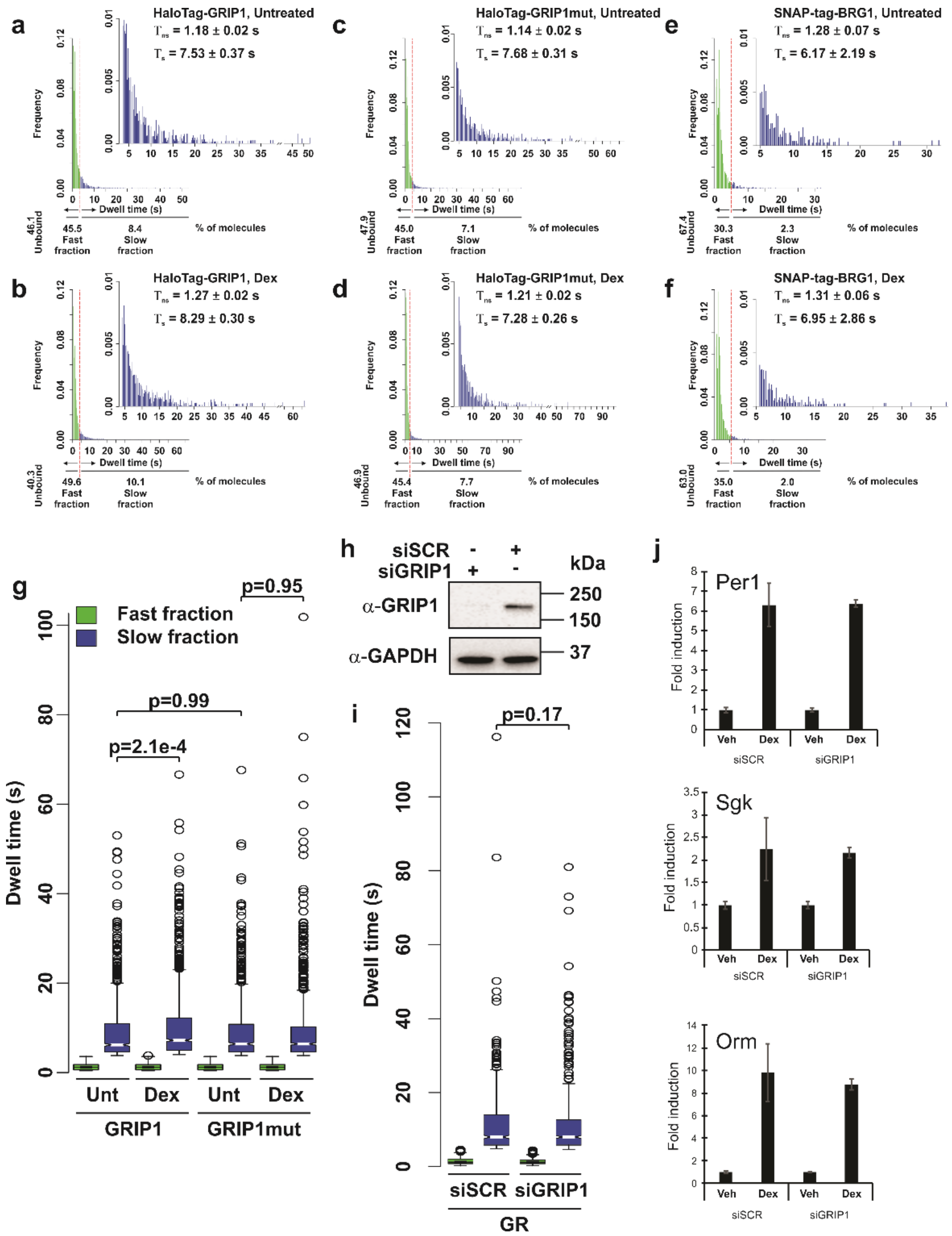

Supplementary Figure 4. **HaloTag-GRIP1, HaloTag-GRIP1mut, and SNAP-tag-BRG1 Single-molecule tracking data.** Histograms represent the distribution of residence times. Red dashed line separates the fast short-lived fraction (green bars) from the slow long-lived fraction (blue bars). Expanded view represents slow long-lived fraction of the residence time distribution. The average residence time of fast short-lived (T_{ns}) and slow long-lived (T_s) fraction are presented in the histogram. The percentage of molecules unbound, bound at the fast short-lived fraction, and bound at the slow long-lived fraction is presented below the histogram for GRIP1 untreated (a), GRIP1 Dex treated (b), GRIP1m untreated (c), GRIP1m Dex treated (d), BRG1 untreated (e), and BRG1 Dex treated (f). g-l. Data represents collected tracks (black circles) showing the single-molecule residence times fitted to a single- (blue line) or double-exponential (red line) decay model. Expanded view represents the single-molecule residence time fitted to a single- (blue line) or double-exponential (red line) decay model with y-axis plotted as a log10. F-test determines the statistical significance of the fit for each treatment condition. (g) Box-plot represents the distribution of dwell times for all molecules in the fast short-lived (green boxes) and slow long-lived (blue boxes) fraction for GRIP1 untreated and Dex treated, GRIP1 mutant (mut), not capable of interacting with GR, untreated and Dex treated cells. Statistical outliers are shown as black circles. p-values represent a Two-sample Kolmogorov Smirnov test defined by the brackets. (h) Immunoblotting against GRIP1 in 3617 cells transfected with control siRNA (siSCR) or siRNA against GRIP1 (siGRIP1). GAPDH antibody was used as a loading control. (i) Box-plot represents the distribution of dwell times for all molecules in the fast short-lived (green boxes) and slow long-lived (blue boxes) fraction for GR in 3617 cells treated with siSCR or siGRIP1. Statistical outliers are shown as black circles. p-values represent a Two-sample Kolmogorov Smirnov test defined by the brackets. (j) mRNA expression of GR target genes, Period 1 (*Per1*), serum and glucocorticoid-regulated kinase (*Sgk*), and orosomucoid (*Orm*) after 1 h Dex treatment in 3617 cells exposed to siSCR or siGRIP1. Bar graph represent mean fold induction ± standard deviation. Data represent at least two biological replicates. Exposure time 10 ms; interval time 200 ms.

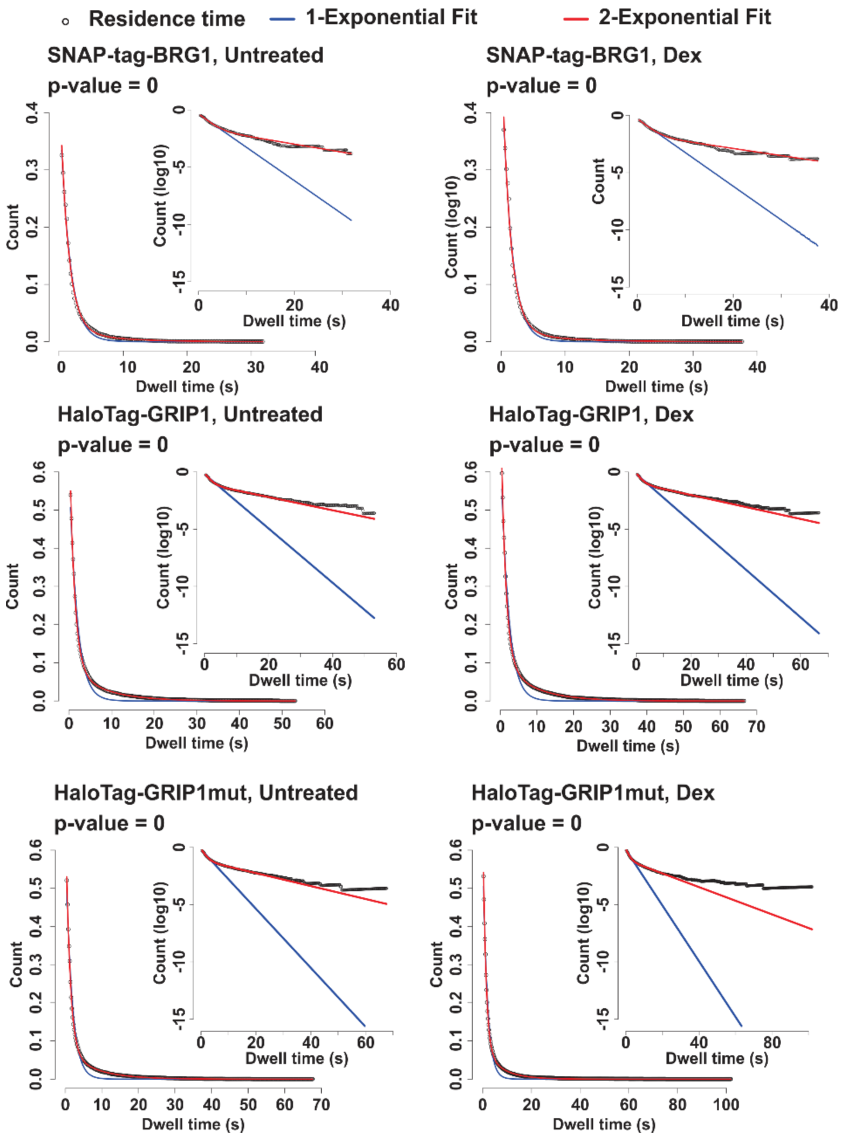

Supplementary Figure 5. **Exponential fitting of HaloTag-GRIP1, HaloTag-GRIP1mut, and SNAP-tag-BRG1 Single-molecule tracking data.** Indicated data represents collected tracks (black circles) showing the single-molecule residence times fitted to a single- (blue line) or double-exponential (red line) decay model. Expanded view represents the single-molecule residence time fitted to a single- (blue line) or double-exponential (red line) decay model with y-axis plotted as a log10. F-test determines the statistical significance of the fit for each treatment condition. Exposure time 10 ms; interval time 200 ms.

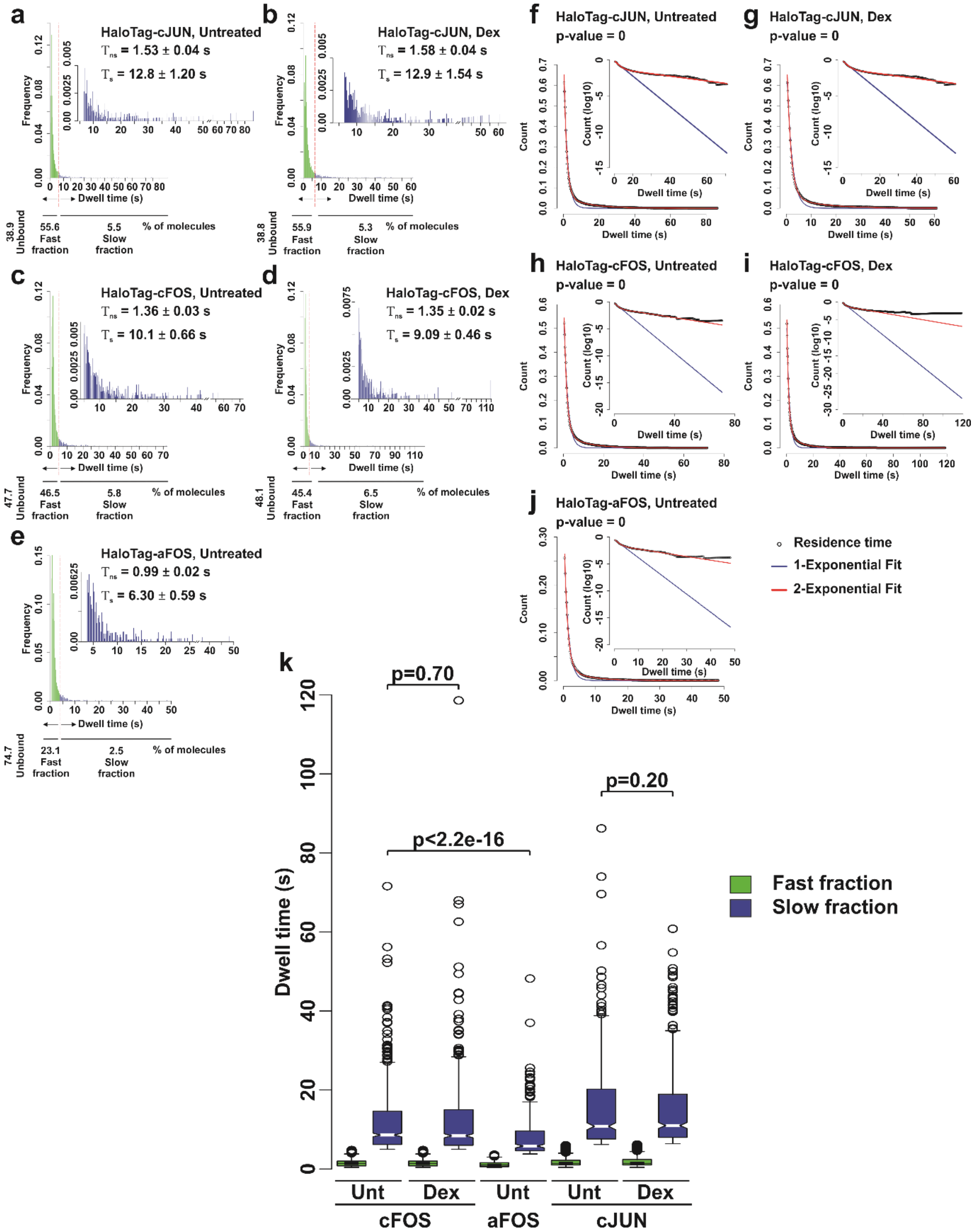

Supplementary Figure 6. **HaloTag-c-JUN, HaloTag-c-FOS, and HaloTag-a-FOS Single-molecule tracking.** Histograms represent the distribution of residence times. Red dashed line separates the fast short-lived fraction (green bars) from the slow long-lived fraction (blue bars). Expanded view represents slow long-lived fraction of the residence time distribution. The average residence time of fast short-lived (T_{ns}) and slow long-lived (T_s) fraction are presented in the histogram. The percentage of molecules unbound, bound at the fast short-lived fraction, and bound at the slow long-lived fraction is presented below the histogram for c-JUN untreated (a), c-JUN Dex treated (b), c-FOS untreated (c), c-FOS Dex treated (d), and a-FOS untreated (e). (f-j) Data represents collected tracks (black circles) showing the single-molecule residence times fitted to a single- (blue line) or double-exponential (red line) decay model. Expanded view represents the single-molecule residence time fitted to a single- (blue line) or double-exponential (red line) decay model with y-axis plotted as a log10. F-test determines the statistical significance of the fit for each treatment condition. (k) Box-plot represents the distribution of dwell times for all molecules in the fast short-lived (green boxes) and slow long-lived (blue boxes) fraction for c-FOS untreated and Dex treated, a-FOS untreated, c-JUN untreated and Dex treated cells. Statistical outliers are shown as black circles. p-values represent a Two-sample Kolmogorov Smirnov test defined by the brackets. Exposure time 10 ms; interval time 200 ms.

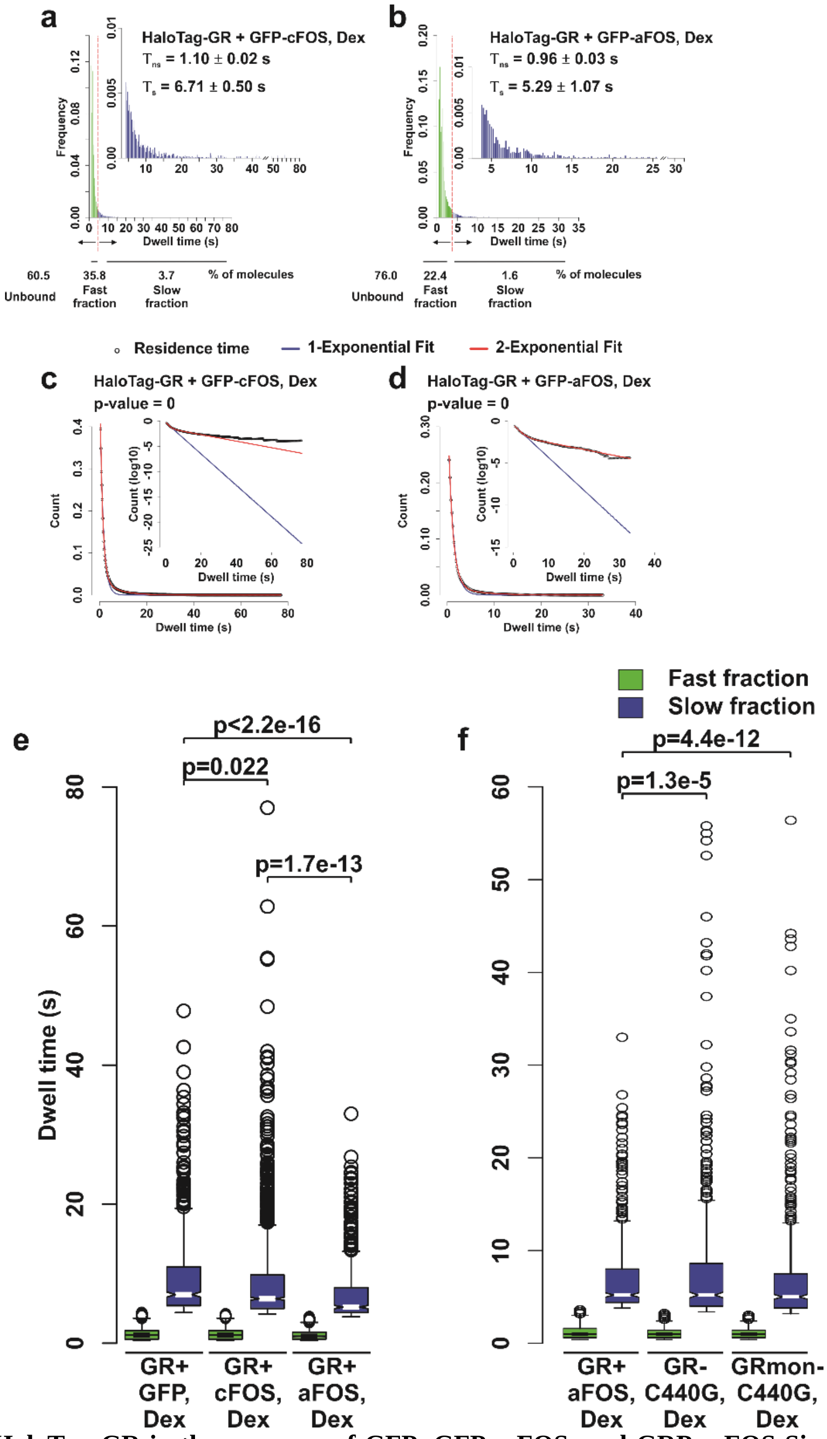

Supplementary Figure 7. **HaloTag-GR in the presence of GFP, GFP-c-FOS, and GRP-a-FOS Single-molecule tracking data.** Histograms represent the distribution of residence times. Red dashed line separates the fast short-lived fraction (green bars) from the slow long-lived fraction (blue bars). Expanded view represents slow long-lived fraction of the residence time distribution. The average residence time of fast short-lived (T_{ns}) and slow long-lived (T_s) fraction are presented in the histogram. The percentage of molecules unbound, bound at the fast short-lived fraction, and bound at the slow long-lived fraction is presented below the histogram for GR+GFP Dex treated (a), GR+GFP-c-FOS Dex treated (b), and GR+GFP-a-FOS Dex treated (c). (d-f) Data represents collected tracks (black circles) showing the single-molecule residence times fitted to a single- (blue line) or double-exponential (red line) decay model. Expanded view represents the single-molecule residence time fitted to a single- (blue line) or double-exponential (red line) decay model with y-axis plotted as a log10. F-test determines the statistical significance of the fit for each treatment condition. (g) Box-plot represents the distribution of dwell times for all molecules in the fast short-lived (green boxes) and slow long-lived (blue boxes) fraction for GR+GFP Dex treated, GR+GFP-c-FOS Dex treated, and GR+GFP-a-FOS Dex treated cells. Statistical outliers are shown as black circles. p-values represent a Two-sample Kolmogorov Smirnov test defined by the brackets. (h) Box-plot represents the distribution of dwell times for all molecules in the fast short-lived (green boxes) and slow long-lived (blue boxes) fraction for GR+GFP-aFOS Dex treated, GRC440G Dex treated, and GRmonC440G Dex treated cells. Statistical outliers are shown as black circles. p-values represent a Two-sample Kolmogorov Smirnov test defined by the brackets. Exposure time 10 ms; interval time 200 ms.

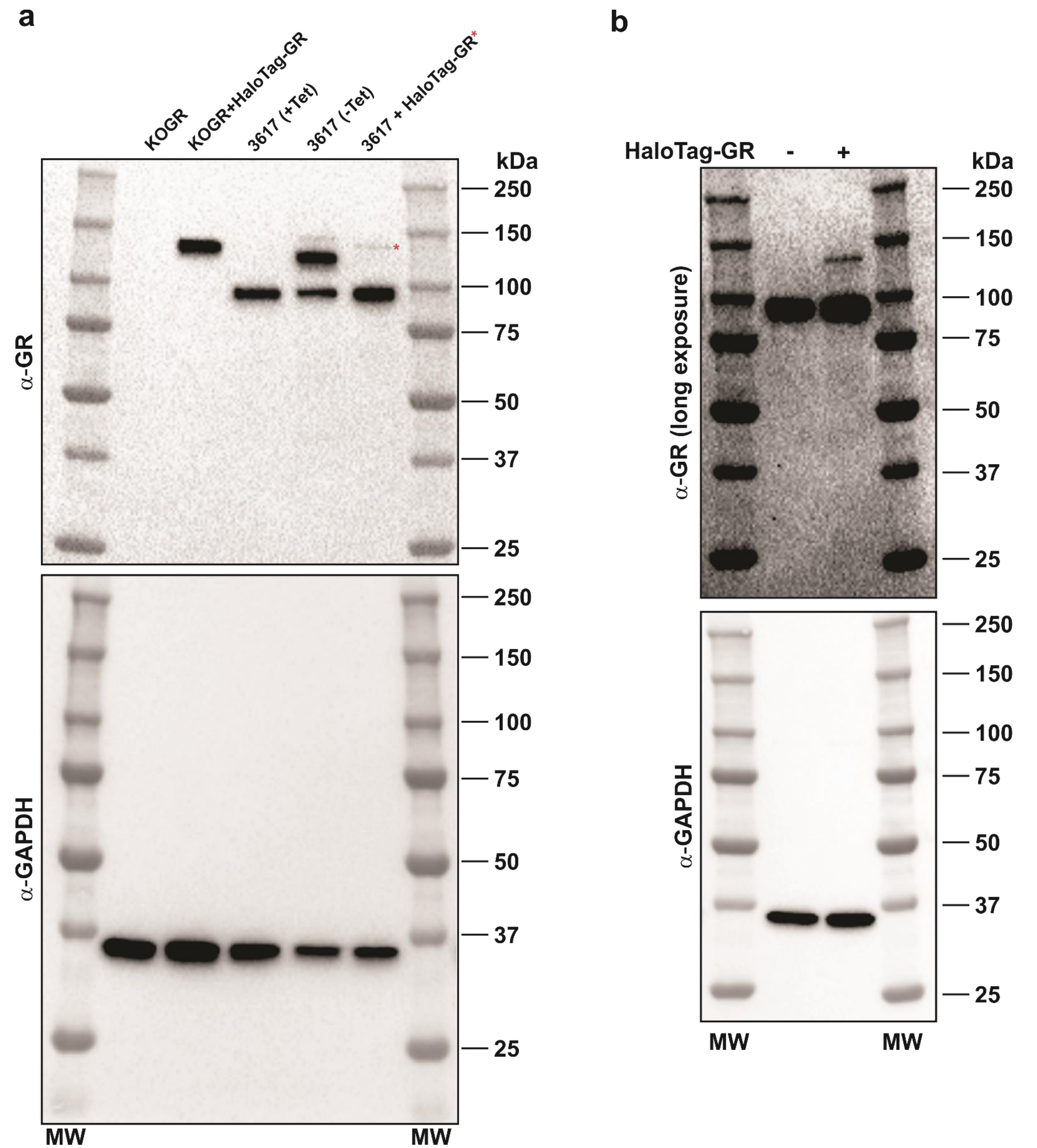

Supplementary Figure 8. Full immunoblots shown in Figure 2a and b. (a) (Upper blot) Immunoblotting against GR shown in Figure 2a. (Lower blot) Immunoblotting against GAPDH shown in Figure 2a. (b) (Upper blot) Immunoblotting against GR shown in Figure 2b. (Lower blot) Immunoblotting against GAPDH shown in Figure 2b. MW, molecular weight marker (Bio-Rad Precision Plus Protein WesternC Standards).

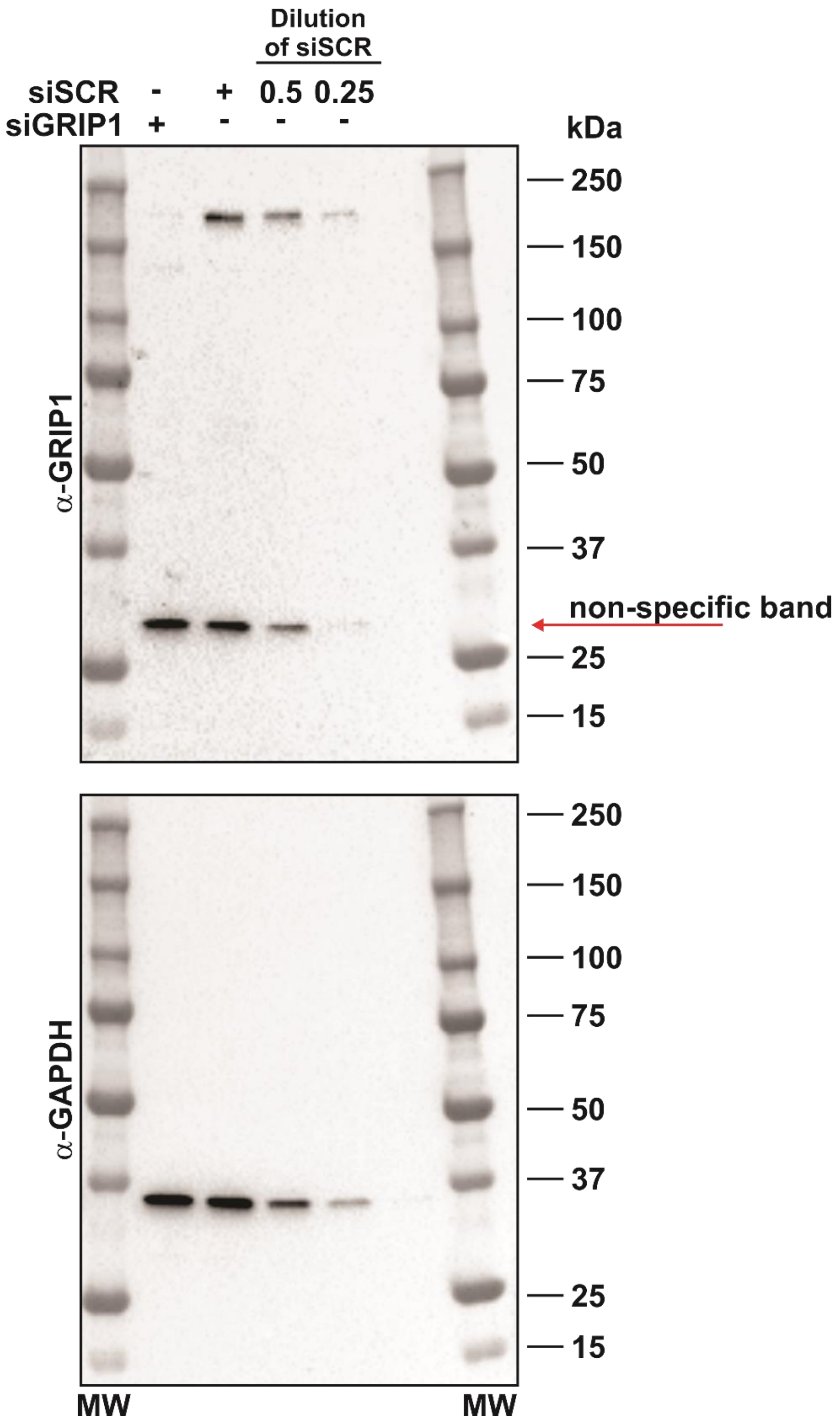

Supplementary Figure 9. Full immunoblots shown in Supplementary Figure 4h. (Upper blot) Immunoblotting against GRIP1 shown in Supplementary Figure 4h. Non-specific ban indicated with red arrow. (Lower blot) Immunoblotting against GAPDH shown in Supplementary Figure 4h. MW, molecular weight marker (Bio-Rad Precision Plus Protein WesternC Standards). Full immunoblots also show dilution of siSCR sample to half (0.5) or to quarter (0.25) of the full sample.

Supplementary Table 1
Photobleaching kinetics

Factor	Exposure time (ms)	Interval time (ms)	kB	Mean survival time (s)	Mean number of frames survived	Half-life of molecule (s)	90th Percentile of survival time (s)	90th Percentile of number of frames survived
HaloTag-GR	10	30	0.077611224	12.88473	429.4911	8.931017	29.66819698	988.9399
HaloTag-GR	10	100	0.05779047	17.30389	173.0389	11.994143	39.84368171	398.4368
HaloTag-GR	10	150	0.037185388	26.89228	179.2819	18.640310	61.92177106	412.8118
HaloTag-GR	10	200	0.026757192	37.37313	186.8656	25.905079	86.05481054	430.2741
HaloTag-GR	10	250	0.021859331	45.74706	182.9882	31.709442	105.33648714	421.3459
HaloTag-GR	10	400	0.013349792	74.90754	187.2688	51.921947	172.48097444	431.2024
HaloTag-GR	10	800	0.006760098	147.92685	184.9086	102.535081	340.61416710	425.7677
HaloTag-GR	10	1000	0.006310875	158.45662	158.4566	109.833762	364.85986087	364.8599
HaloTag-GR	10	1500	0.003385584	295.37003	196.9134	204.734905	680.11463330	453.4098
HaloTag-GR	10	2000	0.001326314	753.96923	376.9846	522.611647	1736.07831434	868.0392
HaloTag-GR	10	2500	0.000983132	1017.15786	406.8631	705.040101	2342.09251992	936.8370

The table shows the photobleaching decay rate, kB, obtained from fitting the number of particles in each frame to a single exponential decay, mean survival time, half-life of molecules, and the time after which only 10% (90th Percentile) of the particles survive for data in Figure 3. For more details, see Methods. The 90th percentiles of particle survival for each interval provides an upper limit on residence time that could be observed for that interval. For the longest time-lapse intervals of 2000 and 2500 ms, these values indicate that it would have been possible to extract a second bound population, with longer residence time up to 1700 and 2300 s, respectively, if they existed.
